# Predicting environmental chemical factors associated with disease-related gene expression data

**DOI:** 10.1186/1755-8794-3-17

**Published:** 2010-05-06

**Authors:** Chirag J Patel, Atul J Butte

**Affiliations:** 1 Department of Pediatrics, Stanford University School of Medicine, Stanford, CA 94305, USA; 2 Center for Biomedical Informatics Research, Department of Medicine, Stanford University School of Medicine, Stanford, CA 94305, USA; 3 Lucile Packard Children's Hospital, 725 Welch Road, Palo Alto, CA 94304, USA

## Abstract

**Background:**

Many common diseases arise from an interaction between environmental and genetic factors. Our knowledge regarding environment and gene interactions is growing, but frameworks to build an association between gene-environment interactions and disease using preexisting, publicly available data has been lacking. Integrating freely-available environment-gene interaction and disease phenotype data would allow hypothesis generation for potential environmental associations to disease.

**Methods:**

We integrated publicly available disease-specific gene expression microarray data and curated chemical-gene interaction data to systematically predict environmental chemicals associated with disease. We derived chemical-gene signatures for 1,338 chemical/environmental chemicals from the Comparative Toxicogenomics Database (CTD). We associated these chemical-gene signatures with differentially expressed genes from datasets found in the Gene Expression Omnibus (GEO) through an enrichment test.

**Results:**

We were able to verify our analytic method by accurately identifying chemicals applied to samples and cell lines. Furthermore, we were able to predict known and novel environmental associations with prostate, lung, and breast cancers, such as estradiol and bisphenol A.

**Conclusions:**

We have developed a scalable and statistical method to identify possible environmental associations with disease using publicly available data and have validated some of the associations in the literature.

## Background

The etiology of many diseases results from interactions between environmental factors and biological factors [1]. Our knowledge regarding interaction between environmental factors, such chemical exposure, and biological factors, such as genes and their products, is increasing with the advent of high-throughput measurement modalities. Building associations between environmental and genetic factors and disease is essential in understanding pathogenesis and creating hypotheses regarding disease etiology. However, it is currently difficult to ascertain multiple associations of chemicals to genes and disease without significant experimental investment or large-scale epidemiological study. Use of publicly-available environmental chemical factor and genomic data may facilitate the discovery of these associations.

We desired to use pre-existing datasets and knowledge-bases in order to derive hypotheses regarding chemical association to disease without upfront experimental design. Specifically, we asked what environmental chemicals could be associated with gene expression data of disease states such as cancer, and what analytic methods and data are required to query for such correlations. This study describes a method for answering these questions. We integrated publicly available data from gene expression studies of cancer and toxicology experiments to examine disease/environment associations. Central to our investigation was the Comparative Toxicogenomics Database (CTD) [2], which contains information about chemical/gene/protein interactions and chemical/gene/disease relationships, and the Gene Expression Omnibus (GEO) [3], the largest public gene expression data repository. Information in the CTD is curated from the peer-reviewed literature, while gene expression data in GEO is uploaded by submitters of manuscripts.

Most approaches to date to associate environmental chemicals with genome-wide changes can be put into 2 categories. These approaches either 1.) have tested a small number of chemicals on cells and measured responses on a genomic scale, or 2.) used existing knowledge bases, such as Gene Ontology, to associate annotated pathways to environmental insult.

The first method involves measuring physiological response on a gene expression microarray. This approach allows researchers to test chemical association on a genomic scale, but the breadth of discoveries is constrained by the number of chemicals tested against a cell line or model organism. These experiments are not intended for hypothesis generation across hundreds of potential chemical factors with multiple phenotypic states. Only a few chemicals can be tractably tested for association to gene activity [4,5], or disease on cell lines [6], or on model organisms, including rat and mouse [7]. In rare cases, this approach has reached the level of a hundred or thousand chemical compounds, such as the Connectivity Map, developed by Lamb, Golub, and colleagues [8], which attempts to associate drugs with gene expression changes. After measuring the genome-wide effect on gene expression after application of hundreds of drugs at various doses, drug signatures are calculated and are then queried with other datasets for which a potential therapeutic is desired. While this has proven to be an excellent system to find chemicals that essentially reverse the genome-wide effects seen in disease, the approach of measuring gene expression and calculating signatures across tens of thousands of environmental chemicals is not always feasible or scalable. Although other data-driven approaches have been described [9], few have given insight into external causes of disease.

A second approach has been to use knowledge bases, such as Gene Ontology [10] to aid in the interpretation of genomic results. For example, Gene Ontology analysis of a cancer experiment might elucidate a molecular mechanism related to an environmental chemical. Unfortunately, there is still a lack of methodology to derive hypotheses for environmental-genetic associations in disease pathogenesis, as Gene Ontology and general gene-set based approaches have limited information on environmental chemicals.

In contrast to the previous approaches, we claim that the integration of pre-existing data and knowledge bases can derive hypotheses regarding the association of chemicals to gene activity and disease from multiple datasets in a scalable manner. Gohlke et al have proposed an approach to predict environmental chemicals associated with phenotypes also using knowledge from the CTD [11]. Their method utilizes the Genetic Association Database (GAD) [12] to associate phenotypes to genetic pathways and the CTD to link pathways to environmental factors. This method has proved its utility, allowing for production of hypotheses for chemicals associated with diseases categorized as metabolic or neuropsychiatric disorders. However, in its current configuration, their method is dependent on the GAD, which contains statically annotated phenotypes in relation to genes containing variants; such DNA changes are not likely to be reflective of molecular profiles of tissues being suspected for environmental influence. Unlike this method, our proposed approach is tissue- and data-driven in that the phenotype is determined by the individual measurements of gene expression in cells and tissues, allowing for the dynamic capture of phenotypes.

The approach we propose here is agnostic to experiment protocol, such as cell line or chemical agent tested, and provides for a less resource-intensive screening of chemicals to biologically validate. Our methodology essentially combines the best features of these current approaches. We start by compiling "chemical signatures" in a scalable way using the CTD. These chemical signatures capture known changes in gene expression secondary to hundreds of environmental chemicals. In a manner similar to how Gene Ontology categories are tested for over-representation, we then calculate the genes differentially expressed in disease-related experiments and determine which chemical signatures are significantly over-represented. We first verified the accuracy of our methodology by analyzing microarray data of samples with known chemical exposure. After these verification studies yielded positive results, we then applied the method to predict disease-chemical associations in breast, lung, and prostate cancer datasets. We validated some of these predictions with curated disease-chemical relations, warranting further study regarding pathogenesis and biological mechanism in context of environmental exposure. Our method appears to be a promising and scalable way to use existing datasets to predict environmental associations between genes and disease.

## Methods

### Method to Predict Environmental Associations to Gene Expression Data

**Figure 1:**
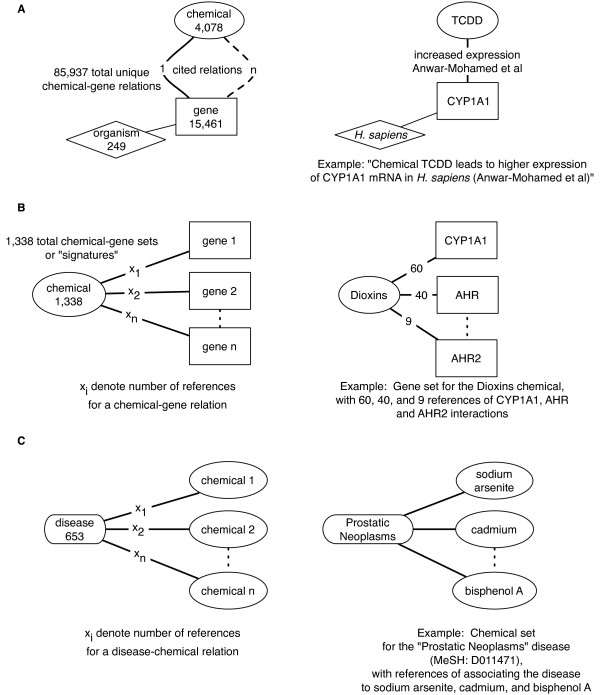
**Prediction database creation based on the Comparative Toxicogenomics Database (CTD)**. A.) The CTD contained 85,937 total unique chemical-gene relations over 4,078 chemicals and 15,461 genes. Each relation had one or more citations of support. An example hypothetical relation, "*TCDD *lead to *higher expression of CYP1A1 *mRNA in *H. sapiens *as shown in *Anwar-Mohamed et al*" is seen on the right panel. B.) Creation of chemical-gene set relations. Each chemical-gene relation had a number of citations of support, x_i_. For each chemical, we constructed a gene set, or "signature" from the individual chemical-gene relations. We filtered out signatures that had at least 5 genes in the set, leaving a total of 1,338 chemical-gene sets. An example of one chemical-gene set is seen on the right panel of B: the genes *CYP1A1*, *AHR, AHR2 *are shown to have multiple citations for the relation, 60, 40, and 9 respectively.

The Comparative Toxicogenomics Database (CTD) includes manually-curated, cross-species relations between chemicals and genes, proteins, and mRNA transcripts [13]. We downloaded the knowledge-base spanning 4,078 chemicals and 15 461 genes and 85 937 relationships between them in January 2009. An example of a relationship in the CTD is "Chemical TCDD results in higher expression of CYP1A1 mRNA as cited by Anwar-Mohamed et al. in *H. sapiens*" (demonstrated in Figure 1A). The median, 70^th^, and 75^th ^percentile of the number of genes related to a chemical is 2, 5, 7 respectively.

With the single gene, single chemical relationships, we created "chemical signatures", or gene sets associated with each chemical (Figure 1B). Gene sets were created from gene-expression relations spanning 249 species, but most relations came from *H. sapiens, M. musculus, R. norvegicus*, and *D. rerio*. We eliminated chemical-gene sets that had less than 5 genes in the set. This step yielded a total of 1,338 chemical-gene sets.

The CTD also contains curated data regarding the association of a diseases to chemicals. These associations are either shown in an experimental model physiological system or through epidemiological studies. We used these curated associations to validate our predicted factors associated to disease. There are 3,997 diseases-chemical associations in the CTD, consisting of 653 diseases (annotated by unique MeSH terms) and 1,515 chemicals (Figure 1C). The median, 70^th^, and 75^th ^, and 80^th ^percentile of the number of curated chemicals per disease is 2, 3, 4, and 5 respectively.

**Figure 2:**
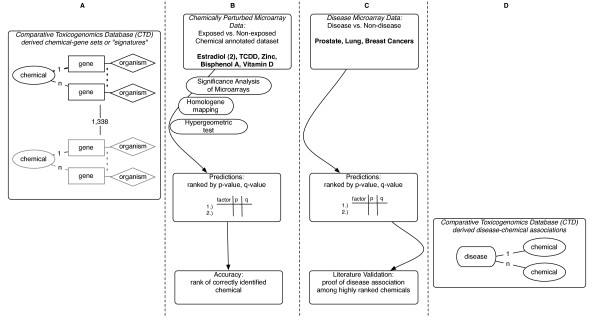
**Predicting environmental chemical association to gene expression datasets**. A.) A representation of the 1338 chemical-gene sets in our prediction database. B.) For the validation step, we conducted SAM to find genes whose expression was altered in each of our datasets. We then mapped the differentially expressed genes to corresponding extra-species genes in our database by using Homologene. For each chemical-gene set signature, we conduct a hypergeometric test for enrichment and ranked each result by p-value. C.) We applied the approach used in B to predict chemical association to prostate, breast, and lung cancer data and validated these results with curated disease-chemical annotations from the CTD represented in D.). D.) Representation of the curated disease-chemical associations in the CTD.

We built a system to test whether genes significantly differentially expressed within a gene expression dataset could be associated with our calculated chemical signatures (Figure 2A). We conducted two phases of analysis in this study. The first phase was a verification one, testing whether the method could accurately predict known chemical exposures applied to samples (Figure 2B). Our input for this first phase were gene expression datasets of chemically-exposed samples and unexposed control samples, and our output were lists of chemicals predicted to be associated with each dataset. The second investigation phase involved predicting chemicals associated with cancer gene expression datasets (Figure 2C). Our input for this second phase were gene expression datasets of cancer samples and control samples, and our output were lists of chemicals predicted to be associated with the dataset. We attempted to validate these findings further by using curated disease-chemical relations (Figure 2D). Finally, we attempted to group our chemical predictions associated with cancer dataset by PubChem-derived BioActivity similarity measures, seeking further evidence of potential underlying mechanism or similar modes of action between chemicals.

We used Significance Analysis of Microarrays (SAM) software to select differentially expressed genes from a microarray experiment [14]. The FDR for SAM for all of our predictions were controlled up to a maximum of 5 to 7% in order to reduce false associations.

We mapped microarray annotations to other corresponding representative species, *H. sapiens, M. musculus*, and *R. norvegicus *using Homologene [15]. In the CTD, gene identifiers were commonly associated with *H. sapiens*; however, some are mapped to specific organisms, such as *M. musculus and R norvegicus*. Most mappings in the CTD are among these 3 organisms. By mapping our expression annotation to these organisms, we ensured gene compatibility with a large portion of the CTD.

We checked for enrichment of differentially expressed genes among our 1,338 chemical-gene sets with the hypergeometric test. To account for multiple hypothesis testing, we computed the q-value, or false discovery rate for a given p-value, by using 100 random resamplings of genes from the microarray experiment and testing each of these random resamplings for enrichment against each of the 1,338 chemical-gene sets. This methodology is similar to the q-value estimation method described in "GoMiner", a gene ontology enrichment assessment tool [16]. We assessed a positive prediction for those that had exceeded a certain p-value and q-value threshold in our list of 1,338 tested associations. All analyses were conducted using the R statistical environment [17].

### Method Verification Phase

For our verification phase, we surveyed publicly available data from the Gene Expression Omnibus (GEO) for experiments in which sets of samples exposed to chemicals were compared with controls. We found and used six datasets in the validation phase. Set 1 included GSE5145 (3 study samples and 3 controls) in which *H. sapiens *muscle cell samples were exposed to Vitamin D [18]. Set 2 was GSE10082 (6 study samples and 5 controls) in which wild-type *M. musculus *were exposed to tetradibenzodioxin (TCDD) [19]. Set 3 was GSE17624 in which *H. sapiens *Ishikawa cells (4 study samples and 4 controls) were exposed to high doses of bisphenol A (no reference). Set 4 was GSE2111 in which *H. sapiens *bronchial tissue (4 study samples and 4 controls) were exposed to zinc sulfate [20]. The CTD had some chemical-gene relations based on this dataset; we removed these relations prior to computing the predictions for this dataset. Set 5 was GSE2889 in which *M. musculus *thymus tissues (2 study samples and 2 controls) were exposed to estradiol [21]. Finally, set 6 was GSE11352 in which *H. sapiens *MCF-7 cell line was exposed to estradiol at 3 different time points [22]. In all cases except for set 6, we treated SAM analysis as unpaired t-tests; for set 6, we used the time-course option in SAM. See Additional File 1 for the number of differentially expressed genes found for each dataset along with their median false discovery rate (Additional file 1, Supplementary Table S1).

### Predicting Environmental Factors Associated with Disease-related Gene Expression Data Sets: Prostate, Lung, and Breast Cancer

We found previously measured cancer gene expression datasets to identify potential environmental associations with cancer. We used measurements from human prostate cancer from GSE6919 [23,24], lung cancer from GSE10072 [25], and breast cancer from GSE6883 [26]. We conducted all SAM analyses using an unpaired t-test between disease and control samples. Additional File 1 shows the number of differentially expressed genes measured for each dataset along with the level of FDR control (Additional file 1, Supplementary Table S2).

We deliberately chose cancer datasets that used a different population of controls rather than normal tissues from the same patients. The prostate cancer dataset (GSE6919) consisted of 65 prostate tissue cancer samples and 17 normal prostate tissue samples as controls.

The lung cancer dataset (GSE10072) consisted of two patient groups: non-smokers with cancer (historically and currently), and current smokers with cancer. We conducted the predictions on these groups separately. The cancer-non smoker group consisted of 16 samples and the cancer-smoker group had 24 samples. The control group consisted of 15 samples.

The breast cancer dataset (GSE6883) consisted of two distinct cancer sub-groups: non-tumorigenic and tumorigenic. As with the lung cancer data, we conducted our predictions on these groups separately. The non-tumorigenic group consisted of three samples and the tumorigenic group had six samples. The control group contained three samples.

We then validated our highly ranked factor predictions with disease-chemical knowledge from the CTD. In particular, we determined if the highly significant chemicals in our prediction list included those that had curated relationship with cancer in the CTD (disease-chemicak relation). This step was similar to measuring association to chemicals via enriched gene sets using the hypergeometric test as described above. We used curated factors associated with Prostatic Neoplasms (MeSH ID: D011471), Lung Neoplasms (D008175), and Breast Neoplasms (D001943), to validate our predictions generated with the prostate cancer, lung cancer, and breast cancer datasets respectively. Further, we assessed the validation by computing the actual number of false positives and true negatives. To compute this number, we assessed whether the prediction list was enriched for chemicals associated with any of the other diseases in the CTD at a higher significance level than the true disease; for this test, we chose diseases that had at least 5 chemical associations, a total of 141 diseases. As an example, to assess the false positive rate for the prostate cancer (MeSH ID: D011471) predictions, we determined the curated enrichment of our predictions for all 140 other disease-chemical sets and counted the number of diseases that had a lower p-value than that computed for D011471.

### Clustering Significant Predictions By PubChem-derived Biological Activity

Chemical-gene sets derived from the CTD are but one representation of how a chemical might affect biological activity. Biological activity of chemicals may also be derived from high-throughput, in-vitro chemical screens such as those archived in PubChem [27,28]. Specifically, the PubChem database provides a large number of phenotypic measurements (or "BioAssays") for many of the chemicals we predicted for cancer. In addition, PubChem provides tools to compare BioAssay measurements for different chemicals. Quantitative and standardized BioAssay measurements (normalized "scores") allow comparison of biological activities of chemicals and derivation of biological activity similarity between chemicals. For example, PubChem represents the biological activity of a compound through a vector of BioAssay scores and assembles a bioactivity similarity matrix between each pair of chemicals with this data.

**Figure 3:**
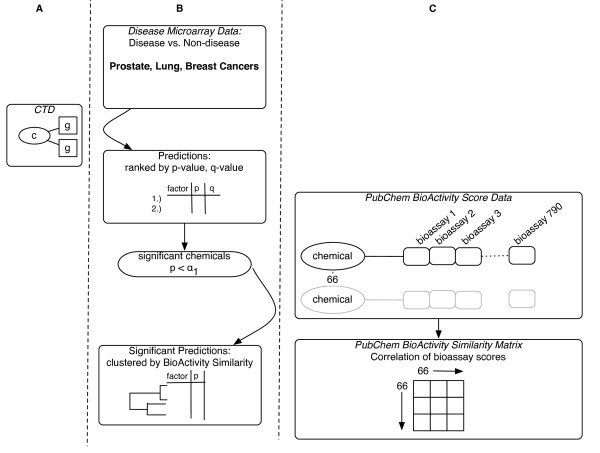
**Clustering chemical prediction lists by biological activity archived in PubChem**. A.) A representation of the CTD and chemical-gene sets as shown in detail in Figure 2. B.) Prediction of the chemicals associated to each cancer dataset using chemical-gene sets from the CTD. We selected highly significant chemical predictions for each cancer and clustered these chemicals by their "Bioactivity" similarity as defined and computed in PubChem. C.) Within PubChem, each of these chemicals has a vector of standardized BioAssay scores. PubChem had 790 BioAssay scores for 66 of our significant predictions. The PubChem BioActivity similarity tool uses these vectors of scores to computes the biological activity similarity for each pair of chemicals and similarity is represented as a matrix.

We sought further external evidence of the relevance of the predicted chemicals though comparison of their patterns of PubChem-sourced biological activity (Figure 3). First, we produced a list of chemical predictions for each cancer dataset as described above (Figure 2, 3A, and 3B) and submitted our list of chemicals to PubChem for activity comparison (Figure 3C). Finally, we observed patterns of correlation between PubChem-derived biological activities of the compounds to their chemical-gene set association significance by clustering the chemicals in the prediction list by their biological activity.

## Results

We implemented a method to predict a list of environmental factors associated with differentially expressed genes (Figure 2). The method is centered on chemical-gene sets that are derived from single curated chemical-gene relationships in the CTD. We determine whether the differentially expressed genes are associated to a chemical by assessing if the expressed genes are enriched for a chemical-gene set, or contain more genes from the chemical-gene set than expected at random using the hypergeometric test. We applied this method in two phases, the first a verification phase in which we sought to rediscover known exposures applied to samples, and a query phase, in which we sought to find factors associated with cancer gene expression datasets. We refer to significant chemical-gene set associations to gene expression data as "associations" or "predictions" in the following.

### Verification Phase

We first applied our method to gene expression data from experiments in which samples were exposed to specific chemicals, reasoning that if our method could identify these known chemical exposures, we could use the method to predict chemicals that may have perturbed gene expression in unknown experimental or disease conditions. Our goal was to determine where a gene expression-altering chemical might lie in the range of significance rankings applied by the prediction method.

We applied our method on datasets that measured gene expression after exposure to vitamin D, tetrachlorodibenzodioxin (TCDD), bisphenol A, zinc, and estradiol (2 datasets) on different tissue types (Additional File 1, Supplementary Table S1). Table 1 shows the results of our predictions along with a subset of genes in the chemical-gene set that were differentially expressed.

**Table 1:** Chemical Prediction Results from the Verification Phase.

Actual Chemical Exposure (GEO accession)	Chemicals Predicted	Hypergeometric P-value	Rank (Percentile)	q-value	Relevant Genes Expressed
Vitamin D3 on *H. sapiens *muscle cells (GSE5145)	Calcitriol	1 × 10^-23^	1 (100)	0	VDR (25), CYP24A1 (14)

TCDD on *M. musculus *(GSE10082)	TCDD	2 × 10^-15^	3 (99)	0	CYP1A1 (59), CYP1B1 (15), AHRR(6), CYP1A2 (14)

Bisphenol A on *H. sapiens *Ishikawa cells (GSE17624)	Bisphenol A	1 × 10^-6^	15 (99)	0	ESR1(31), ESR2(7), S100G (6)

Zinc sulfate on *H. sapiens *bronchial tissue (GSE2111)	Zinc sulfate	3 × 10^-3^	15 (99)	0.04	SLC30A1 (3), MT1F(2), MT1G(2)

Estradiol on *M. musculus *thymus (GSE2889)	Estradiol	5 × 10^-3^	17 (99)	0.08	C3(6), LPL (4), CTSB (2)

Estradiol on *H. sapiens *MCF7 cell line (GSE11352)	Estradiol	5 × 10^-3^	19 (99)	0.08	ISG20 (2), MGP (2), SERPINA1 (2)

Each row represents a gene expression dataset and relevant prediction and ranking. The first column specifies the gene expression dataset, the 2^nd ^column the actual exposure applied to the samples for the gene expression set. The 3^rd ^and 4^th ^columns represent the hypergeometric p-value for chemical-gene set enrichment along with the rank of the chemical in the prediction list. The 5^th ^column shows the 5^th ^percentile of the ranking derived from 100 random samplings of genes from the gene expression dataset. The 6^th ^column show notable genes expressed in the chemical-gene set along with the number of references the chemical-gene relation in the CTD.

We were able to satisfactorily predict the exposures applied to the gene expression datasets. We ascertained a positive prediction if the exposure had a relatively high ranking (low p-value for enrichment) and if the q-value was lower than 0.1. For the datasets measuring expression after exposure to Vitamin D, calcitriol, a type of vitamin D, was ranked first in the list (p = 10^-23^, q = 0). Similarly, TCDD was predicted third in its respective list (p = 10^-15^, q = 0). The other exposures ranked within the top percentile, ranging from 15 to 19; the lower bound of p-values were between 10^-6 ^and 0.01 and q-values less than 0.1. We reasoned that we could detect true associations between environmental chemicals and gene expression phenotypes provided they met these significance thresholds.

### Predicting Environmental Chemicals Associated with Cancer Data Sets

We applied our prediction methods to datasets measuring the gene expression for prostate, breast, and lung cancers. In particular, we computed predictions for prostate cancer from primary prostate tumor tissue, lung adenocarcinomas from lung tissue from non-smoking individuals, and non-tumorigenic breast cancer cells grown in mouse xenografts. Additional File 1 shows predictions for related data on tumorigenic breast cancer and smoker lung cancer samples (Additional file 1, Supplementary Tables S3 and S4). To validate and select specific predictions from our ranked list of 1,338 environmental chemicals, we measured how enriched top-ranking chemicals were for annotated disease-chemical citations in for diseases of interest ("Prostate Neoplasms", "Breast Neoplasms", and "Lung Neoplasms"). To call a positive chemical association or prediction to disease phenotype, we used p-value thresholds similar to what we observed during the verification phase (α ≤ 10^-4^, 0.001, 0.01) along with q-values as low as possible, specifically less than 0.1. For comparison, we also used the typical p-value threshold of 0.05.

**Figure 4:**
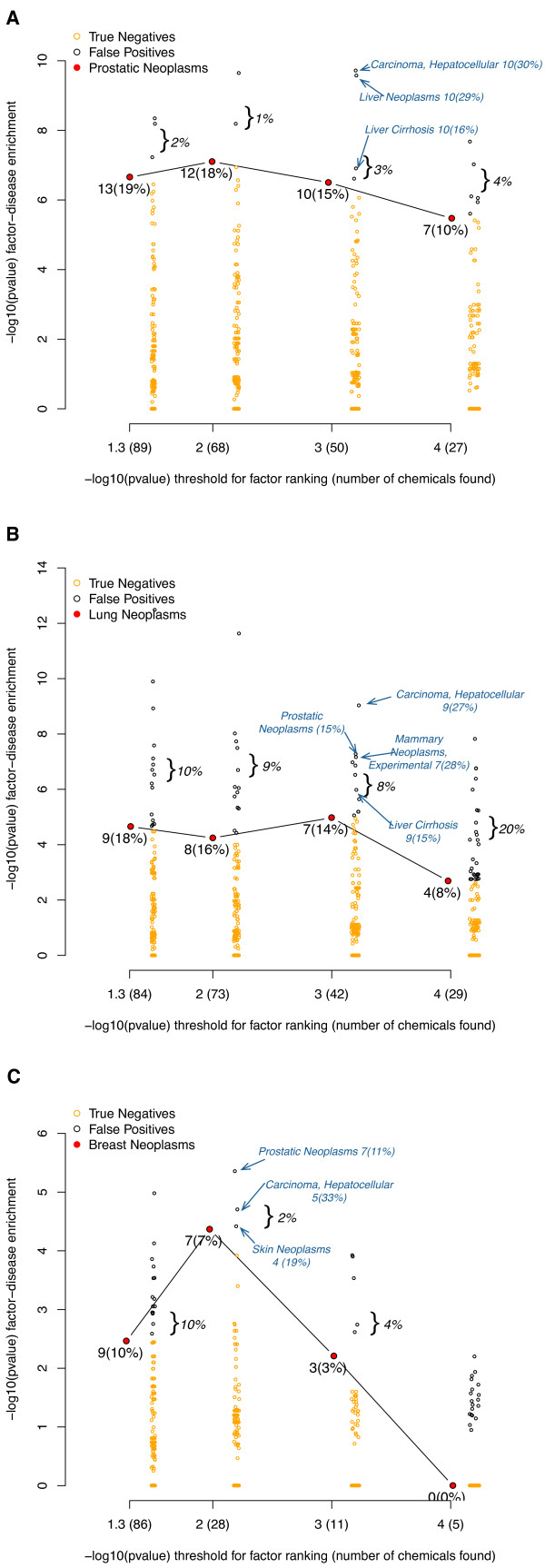
**Curated disease-chemical enrichment versus prediction lists for prostate, lung, and breast cancer datasets**. For a prediction list, we selected chemicals that ranked within α = 10^-4^, 10^-3^, 10^-2^, and 0.05. This -log10(threshold) along with number of total chemicals found (in parentheses) for each threshold is seen on the x-axis of each figure. We tested if these highly ranked chemicals found under each threshold were enriched for chemicals that had known curated association with the cancer in question. The -log10(p-value) for this enrichment is seen on the y-axis. The solid round red marker represents the enrichment test for the actual disease for which the predictions were based; the number underneath represents the total number of chemicals found in the prediction list that had a curated association with the disease and the percent found among all curated relations for that disease. We estimated accuracy and precision by computing disease-chemical enrichment for all other diseases; false positives are offset in black and true negatives are in yellow. The false positive rate is bracketed and in italics. Examples of false positives are annotated in blue italics along with the number of chemicals found in the prediction list corresponding to that disease and the percent found among all curated relations for that disease. We computed this validation enrichment for A.) prostate cancer, B.) lung cancer from nonsmokers, and C.) non-tumorigenic breast cancers.

Figure 4 shows the result of the disease validation phase. In all cases, the signficant chemicals contained many of the specific curated disease-chemical relations. For example, if we call chemicals with p-values less than 0.01 as positive predictions, then we were able to capture 18%, 16%, and 7% of all of the curated relationships for prostate, lung, and breast cancers respectively (p = 10^-7^, 10^-4^, and 4 × 10^-5^). We assessed specificity of our list by computing how many curated chemicals we found for all other diseases in the CTD (Figure 4, offset points in orange and black). We achieved false positive rates between 1 to 4% for prostate cancer, 8 to 20% for lung cancer, and 2 to 10% for breast cancer. However, most all of the "false positives" were other types of neoplasms or cancers (Figure 4, examples annotated in italics/arrows). For example, for the lung and prostate cancer predictions at α = 0.001 only 1 disease other than neoplasm or carcinoma was detected: Liver Cirrhosis, Experimental (MeSH ID: MESH:D008325).

For the prostate cancer dataset, we chose a chemical signature association threshold of 0.001 (q ≤ 0.01). Of 1,338 chemicals tested, 50 total were found under this threshold. Of these 50 chemicals predicted, 10 had a curated relation with the MeSH term "Prostate neoplasms". This amounted to prediction of 15% of all CTD curated disease-chemical relations for the Prostatic Neoplasms term (p = 3 × 10^-7^). These chemicals are seen in Table 2 and include estradiol, sodium arsenite, cadmium, and bisphenol A. Also predicted were known therapeutics, including raloxifene, doxorubicin, genistein, diethylstilbestrol, fenretinide, and zinc. We observed that many of the genes detected were well-studied, additional support to our predictions. For example, *ESR2*, *PGR*, and *MAPK1 *had 37, 34, and 14 references respectively citing their activity in the context of estradiol exposure (Table 2, second-to-right column). Second, we observed common occurrence of genes such as *ESR2*, *BCL2*, and *MAPK1*, among some of the gene sets associated with chemicals such as estradiol, raloxifene, sodium arsenite, doxorubicin, diethylstilbestrol, and genistein.

**Table 2:** Prediction of environmental chemicals associated with prostate cancer samples (GSE6919).

Chemical Predicted	Hypergeometric P-value	Rank (percentile)	q-value	Relevant genes in set (number of references)	Citations
Estradiol	4 × 10^-10^	5 (99)	0	ESR2(37), PGR(34), MAPK1(14)	[37]

Raloxifene	1 × 10^-9^	6 (99)	0	ESR2(6), IGF1(5), BCL2(4)	[38]

Sodium arsenite	1 × 10^-8^	8 (99)	0	JUN(13), MAPK1(9), CCND1(8), FOS(6)	[30]

Doxorubicin	7 × 10^-7^	11 (99)	0	BCL2(23), MAPK1(14), TNF(10)	[39-42]

Cadmium	6 × 10^-6^	13 (99)	0	MT2A(14), MT1A(12), MT3(11), MT1(6)	[43]

Genistein	3 × 10^-5^	19 (99)	6 × 10^-4^	ESR2(22), PGR (10), MAPK1 (5)	[44-46]

Diethylstilbestrol	3 × 10^-5^	22 (98)	0.001	ESR2(8), FOS(8), HOXA10(4)	[47,48]

Fenretinide	3 × 10^-4^	40 (97)	0.004	BCL2(3), ELF3(2), LDHA(2)	[49]

Bisphenol A	6 × 10^-4^	47 (96)	0.01	PGR(8), ESR2(7), IL4RA(2)	[37]

Zinc	9 × 10^-4^	53 (96)	0.01	MT3(18), MT2A(13), MT1A(11)	[50-53]

Shown in the table are a subset of the highly ranked chemicals (p < 0.001) that were predicted to have association with prostate cancer gene expression and had evidence of association with the MeSH term "Prostatic Neoplasms" as in the CTD. The 1^st ^column represents the chemical predicted and the 2^nd ^and 3^rd ^columns show the hypergeometric p-value and ranking. The 4^th ^column shows q-value derived from random samples of genes. The 5^th ^column shows the notable genes in the chemical-gene set that were differentially expressed. The 6^th ^column contains references for the prostate cancer and chemical association found from the CTD.

For the lung cancer dataset, we also chose a threshold of 0.001 (q ≤ 0.004). Of 1,338 chemicals tested, 42 were found under this threshold. Of these 42 chemicals, 7 had a cited relation with "Lung neoplasms", 14% of all curated disease-chemical relations for the term (p = 1 × 10^-5^). These chemicals are seen in Table 3. For lung cancer, we observed cited chemicals such as sodium arsenite, vanadium pentoxide, dimethylnitroamine, 2-acetylaminoflourene, and asbestos. Therapeutics observed included doxorubicin and indomethacin. We did not observe common genes represented for different chemical-gene sets, unlike the prostate cancer predictions. Predictions for the smoker-lung cancer samples were similar, resulting in sodium arsenite, dimethylnitrosamine, and vanadium pentoxide, albeit through different differentially expressed genes (Additional File 1, Supplementary Figure S1 and Table 3).

**Table 3:** Prediction of environmental chemicals associated with lung cancer samples (GSE10072).

Chemical Predicted	Hypergeometric P-value	Rank (percentile)	q-value	Relevant genes in set (number of references)	Citations
Doxorubicin	1 × 10^-6^	16 (99)	4 × 10^-4^	CASP3(60), ABCB1(28), BAX(26), BCL2 (23)	[54]

Sodium arsenite	8 × 10^-6^	20 (98)	4 × 10^-4^	JUN(13), NQ01(6), EGR1(6)	[55-57]

Vanadium pentoxide	1 × 10^-5^	24 (98)	6 × 10^-4^	HBEGF(3), CDK7(1), CDKN1B (1), CDKN1C(1)	[58]

Dimethylnitrosamine	6 × 10^-5^	27 (98)	7 × 10^-4^	TGFB1(23), TIMP1(15), PCNA(6)	[31]

Indomethacin	2 × 10^-4^	34 (97)	0.002	BIRC5(3), CDKN1B(2), MMP9(2)	[59-61]

2-Acetylaminofluorene	3 × 10^-4^	36 (97)	0.003	ABCB1(4), ABCG2(4), KRT19(2)	[62]

Asbestos, Serpentine	4 × 10^-4^	39 (97)	0.004	IL6(2), MMP9(2), MMP12(2), PDGFB(2)	[63]

Shown in the table are a subset of the highly ranked chemicals (p < 0.001) that were predicted to have association with lung cancer gene expression (non-smokers) and had evidence of association with the MeSH term "Lung Neoplasms". Columns have similar definitions as Table 2.

For the breast cancer dataset, we chose a threshold of 0.01 (q ≤ 0.08). Of 1,338 chemicals tested, 28 were found under this threshold. Of these 28 chemicals, 7 had a cited relation with "Breast neoplasms", 7% of all curated disease-chemical relations for the disease. These chemicals are seen in Table 4 (p = 4 × 10^-5^). The chemicals predicted included progesterone and bisphenol A. Therapeutics found included indomethacin and cyclophosphamide. There was evidence for both a harmful chemical and a therapeutic for chemicals such as estradiol, genistein, and diethylstilbestrol for breast cancer. Unlike the predictions shown for prostate and lung cancer, the genes utilized in the predictions for breast cancer were not as well studied, with 1 to 3 references for the gene and environment association. We observed some commonality in chemical-gene sets, such as the presence of *IL6 *and *CEBPD *in several of the top chemicals predicted in association to the disease. Similar chemicals were predicted for the tumorigenic breast cancer dataset, such as estradiol and progesterone. However, chemicals not highly ranked in the non-tumorigenic predictions included benzene and the therapies tamoxifen and resveratrol (Additional File 1, Supplementary Figure S2 and Supplementary Table S4).

**Table 4:** Prediction of environmental chemicals associated with breast cancer samples (GSE6883).

Chemical Predicted	Hypergeometric P-value	Rank (percentile)	q-value	Relevant genes in set (number of references)	Citations
Progesterone	2 × 10^-4^	6 (99)	0.01	IL6(3), STC1(3), CEBPD(2)	[64,65]

Genistein	6 × 10^-4^	10 (99)	0.03	CEBPD(1), APLP2(1), MLF1(1)	[66-68]

Estradiol	7 × 10^-4^	11 (99)	0.03	LPL(4), IL6(3), CEBPD(2)	[69-73]

Indomethacin	3 × 10^-3^	17 (99)	0.05	CCDC50(1), BIRC3(1), DNAJB(1)	[74]

Diethylstilbestrol	3 × 10^-3^	18 (99)	0.05	IL6(1), MARCKS(1), MXD1(1), MMP7(1)	[75,76]

Cyclophosphamide	4 × 10^-4^	19 (99)	0.06	IL6(3), MARCKS(1), PSMA5(1)	[77-79]

Bisphenol A	6 × 10^-3^	21 (99)	0.08	CEBPD(1), MLF1(1), DTL(1)	[80]

Shown in the table are a subset of the highly ranked chemicals (p < 0.01) that were predicted to have association with breast cancer gene expression (non-tumorigenic) and had evidence of association with the MeSH term "Breast Neoplasms". Columns have similar definitions as Table 2.

**Figure 5:**
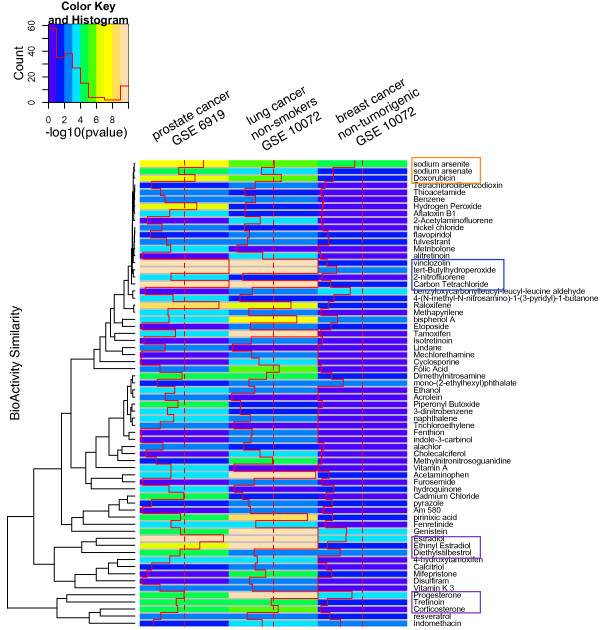
**Chemical predictions for Prostate, Lung, and Breast Cancer datasets clustered by PubChem BioActivity**. Highly significant chemical prediction p-values for the prostate, lung, and breast cancer datasets (p = 0.001, 0.001, 0.01, for the prostate, lung, and breast cancer datasets) are reordered by their BioActivity similarity computed by PubChem. A column represents the cancer analyzed and each cell corresponds to the chemical-gene set association -log10(p-value). Examples of correlation between BioActivity similarity and chemical-gene set significance include the sodium arsenite, sodium arsenate, and Doxorubicin cluster (labeled in orange), the Genistein, Estradiol, Ethinyl Estradiol, and Diethylbisterol and Progesterone, Tretinoin, and Corticosterone clusters (labeled in purple). Other examples of BioActivity similarity and chemical-gene set association include chemicals vinclozolin, tert-Butylhydroperoxide, and Carbon Tetrachloride (outlined in blue).

Some of the chemicals found were common to more than one type of cancer (Figure 5). For example, we predicted chemicals such as sodium arsenite for both prostate cancer and lung cancers, and bisphenol A for both prostate and breast cancers. In some of the cases, the predicted chemical overlap across different cancers are due to the expression of distinct genes for each dataset, highlighting the potential of many possibilities for interaction between environmental chemicals and genes.

### Clustering Significant Predictions by PubChem-derived Biological Activity

We have described a method of generating a list of chemical predictions associated with disease-annotated gene expression datasets and applied the method on gene expression data for several cancers. We have validated a subset of our predictions with evidence from the literature as described above (Tables 2, 3, 4).

We sought further evidence of the biological relevance of our predictions through internal comparison of their potential activity archived in PubChem. Specifically, we expected some degree of correlation between "similar" chemicals and their gene set significance to the cancer datasets. We opted to use PubChem BioActivity to assess chemical similarity, assuming this measure of phenotypic similarity would be representative of underlying biological pathways of action. We picked chemicals that were deemed significant for thresholds used above (p = 0.001, 0.001, 0.01, for the prostate, lung, and breast cancer datasets) for all of the cancer datasets. This resulted in a total of 130 chemicals, 66 of which had BioActivity data in PubChem. The BioActivity similarity for each of the 66 chemicals was computed through 790 BioAssay scores. Figure 5 shows the -log10 of significance for the highest ranked chemical predictions clustered by their BioActivity similarity.

We found some chemicals with similar biological activity profiles in PubChem had similar patterns of chemical-gene set association across the cancer datasets. For example, sodium arsenite, sodium arsenate, and doxorubicin have closely related biological profiles as well as high significance of chemical-gene set association for the prostate and lung cancer data (Figure 5, enclosed in orange box); however, we did not observe other biologically similar chemicals such as Tetradihydrobenzodioxin. On the other hand, we also observed correlation between the biological activity similarity and chemical-gene set association for hormone or steroidal chemicals such as ethinyl estradiol, estradiol, and diethylstilbestrol as well as progesterone and corticosterone (Figure 5, enclosed in purple boxes).

## Discussion

We have developed a knowledge- and data-driven method to predict chemical associations with gene expression datasets, using publicly available and previously disjoint datasets. To our knowledge, there are few methods that generate hypotheses regarding environmental associations with disease from gene expression data. Most current approaches in toxicology have focused on a small number of environmental influences on single or small groups of genes, while current approaches in toxicogenomics have been concentrated on measuring genome-wide responses for a few chemicals [29]. Our prediction method enables the generation of hypotheses in a larger scalable manner using existing data, examining the potential role of hundreds of chemicals over thousands of genome-wide measurements and diseases.

As an example, we found predicted chemicals such as sodium arsenite in its association with prostate and lung cancers, estrogenic compounds such as bisphenol A and estradiol with prostate and breast cancers, and dimethylnitrosamine with lung cancer. Although each has curated knowledge behind the association in the CTD, mechanisms for the action are not well known and call for further study. So far, Benbrahim-Talaa et al have found hypomethylation patterns in the presence of arsenic in prostate cancer cells [30]. Zanesi et al show a potential interaction role of *FHIT *gene and dimethylnitrosamine to produce lung cancers [31]. Evidence of a complex mechanistic action of estrogens, such as estradiol, on breast cancer carcinogenesis has been established [32]; however the role of other estrogenic-like compounds have only recently been studied. For example, bisphenol A has been shown to invoke an aggressive response in cancer cell lines [33], possibly by affecting estrogen-dependent pathways [34]. It is evident that more experimentation is required involving the measurements of exposure-affected proteins and genes and their activation state in cellular models and their relation to the chemical signatures.

An overlap of activity of the same genes induced by different chemicals would suggest a common physiological action by these chemicals. For example, the *ESR2 *and *MAPK1 *genes in the prostate cancer prediction, and the *IL6 *and *CEBPD *in the breast cancer predictions, were associated with several chemicals for each of the diseases. We also found an overlap between chemicals amongst different cancers. This result comes as a result of the correlation in the significant pathways shared by these cancers; however, it may also indicate a need to explore less significant associations in order to find unique and specific gene expression/chemical exposure relationships for a given disease. Furthermore, this result may also indicate a bias of gene and chemical relationships cataloged in the CTD. For example, it could be that genes specific to common cancer-related pathways are those that are well studied, such as *BCL2 *or *ESR2*.

Related to this, we have attempted to show how biological activity, as assayed in a high-throughput chemical screen in PubChem, can be correlated with chemical gene-set associations. Observing a correlation in both PubChem-derived bioactivity in addition to a chemical-gene set association from the CTD provides a way to identify shared modes of action among groups of similar or related chemicals. This data serves to both provide internal validation for list of predicted chemicals acting through similar pathways (such as those induced by estrogen) but also to prioritize hypotheses. For example, we did not find curated evidence in the CTD for association of the chemicals vinclozolin, tert-Butylhydroperoxide, and Carbon Tetrachloride to prostate or lung cancers; however, their similar bioactivity profiles (Figure 5, enclosed in blue box) and high chemical-gene set association calls for further review.

We do acknowledge some arbitrariness in our choice of methods and thresholds; most of these were chosen to show significance in our methodology without adding complexity. We could have chosen any of several alternative approaches to implementing our method; however, predictions made with the Gene Set Enrichment Analysis (GSEA) [35] method during the verification phase were not as sensitive (not shown). Another limitation in our first implementation is that in calculating the chemical signatures associating chemicals with gene sets, we ignored the specific degree of expression change (up or down) encoded in the CTD. We decided not to use this information due to the presence of contradictions (some references may point to an increase of exposure-induced gene expression while another reference might claim the opposite), and other preliminary work suggesting that filtering by the degree of change reduced sensitivity (data not shown). Because of these limitations, direction of association cannot be inferred. Further still, we acknowledge richer and more refined chemical signatures along with further integration with resources like PubChem will need to be built to make the most accurate predictions.

Another issue with querying the microarray data of any experiment is the lack of full sample information to stratify results; for example, different exposures may be associated with a subset of the samples. A related concern includes small sample sizes of some of the datasets used to evaluate the method. For example, the best predictive power was seen the largest dataset (prostate cancer, GSE6919), and the worst with one of the smallest, (breast cancer, GSE6883). Despite this heterogeneity and lack of power, we still arrived at noteworthy and literature-backed findings warranting further study. We also urge that more evaluation must occur with datasets that have a larger number of samples.

Most importantly, we stress that these types of association remain as predictions and hypotheses that need validation and verification. The method presented here is not a substitute for traditional toxicology or epidemiology. These studies are required to provide quantitative and population generalizable estimates of disease risk and dose-response relationships. However, as the space of potential environmental chemicals potentially causing biological effects is large, we suggest that this methodology would give investigators at least some clue where to start the search for environmental causal factors to study in these other modes. Furthermore, predicting a linkage between chemicals, genes, and clinically-relevant disease phenotypes using existing resources falls in agreement with the National Academies' vision of high-throughput efforts to decipher genetic pathways to toxicity [36].

## Conclusion

We have described a novel and scalable method to associate changes in gene expression with environmental chemicals. While we successfully validated our methodology here and provide hypotheses regarding the potential association of chemicals in cancer development, these hypotheses would need to be carefully studied in controlled cellular experiments. Our method is limited by the lack of direction of association and effect size as typically ascertained in traditional toxicological and epidemiological studies; however, the vast number of chemicals that can be tested *in silico *is only limited by the amount of available data. This method is just one of potentially many tools that need to be built to predict environmental associations between genes and disease.

## Abbreviations

CTD: Comparative Toxicogenomics Database; GEO: Gene expression omnibus; GAD: Genetic Association Database; GSEA: Gene Set Enrichment Analysis; DNA: deoxyribonucleic acid; MeSH: Medical Subject Headings; mRNA: messenger RNA; TCDD: 2,3,7,8-Tetrachlorodibenzodioxin (TCDD); SAM: Significance Analysis of Microarrays; FDR: False Discovery Rate.

## Competing interests

The authors declare that they have no competing interests.

## Authors' contributions

AJB conceived of the study and edited the manuscript. CJP carried out the analysis and drafted the manuscript. All authors have read and approved the final manuscript.

## Pre-publication history

The pre-publication history for this paper can be accessed here:

http://www.biomedcentral.com/1755-8794/3/17/prepub

## Supplementary Material

Additional file 1 **Differential gene expression summary information for the verification and query stage and additional lung and breast cancer queries**. Additional file 1 contains information regarding the Significance Analysis of Microarray (SAM) procedure for the verification and query stage, specifically the types of samples analyzed, the median false discovery rate for the analysis, and the number of differentially expressed genes found. Information for the verification stage is in Supplementary Table S1, for the query stage in Supplementary Table S2. We also conducted additional query predictions on gene expression datasets related to the ones described in the main manuscript, specifically on lung cancer smoker samples and tumorigenic breast cancer cell lines. These data are analogous to the Tables 2, 3, 4 in the main manuscript and are seen in Supplementary Tables S3, S4, and S5. Figures analogous to Figure 4 are also seen in Supplementary Figures S1 and S2. Click here for file
