# Supplementary material for: Predicting environmental chemical factors associated with disease-related gene expression data
Source: BMC Med Genomics. 2010 May 6;3:17. doi: 10.1186/1755-8794-3-17 (PMC2880288; doi:10.1186/1755-8794-3-17)
Supplement: Additional file 1 — Differential gene expression summary information for the verification and query stage and additional lung and breast cancer queries. Additional file 1 contains information regarding the Significance Analysis of Microarray (SAM) procedure for the verification and query stage, specifically the types of samples analyzed, the median false discovery rate for the analysis, and the number of differentially expressed genes found. Information for the verification stage is in Supplementary Table S1, for the query stage in Supplementary Table S2. We also conducted additional query predictions on gene expression datasets related to the ones described in the main manuscript, specifically on lung cancer smoker samples and tumorigenic breast cancer cell lines. These data are analogous to the Tables 2, 3, 4 in the main manuscript and are seen in Supplementary Tables S3, S4, and S5. Figures analogous to Figure 4 are also seen in Supplementary Figures S1 and S2. [file 1755-8794-3-17-S1.DOC]

Predicting environmental chemical factors associated with disease-related gene expression data

Chirag J Patel, Atul J Butte

Additional File 1

Title: Differential gene expression summary information for the verification and query stage and additional lung and breast cancer queries

Description: Additional file 1 contains information regarding the Significance Analysis of Microarray (SAM) procedure for the verification and query stage, specifically the types of samples analyzed, the median false discovery rate for the analysis, and the number of differentially expressed genes found. Information for the verification stage is in Supplementary Table S1, for the query stage in Supplementary Table S2.

We also conducted additional query predictions on gene expression datasets related to the ones described in the main manuscript, specifically on lung cancer smoker samples and tumorigenic breast cancer cell lines. These data are analogous to the Tables 2, 3, 4 in the main manuscript and are seen in Supplementary Tables S3, S4, and S5. Figures analogous to Figure 4 are also seen in Supplementary Figures S1 and S2.

All references pertaining to the supplementary information are seen at the end of this document.

**Additional File 1: Supplementary Table S1.**  **Gene expression dataset summary for verification stage.**

| **Dataset** | **Chemical Tested** | **Number of Samples/Controls (tissue type)** | **SAM: median FDR** | **Number of Differentially Expressed Genes / Total** |
| --- | --- | --- | --- | --- |
| GSE5145 [1] | Vitamin D3 | 3/3 (*H.sapiens* muscle) | 0.04 | 805/20555 |
| GSE10082 [2] | TCDD | 6/5 (*M. musculus* injection) | 0.05 | 2066/21863 |
| GSE17624 | Bisphenol A | 4/4 (*H. sapiens* Ishikawa cells)* | 0.04 | 8406/20828 |
| GSE2111 [3] | Zinc sulfate | 4/4 (*H. sapiens* bronchial tissue) | 0.05 | 31/13306 |
| GSE2889 [4] | Estradiol | (*M. musculus* thymus) | 0.07 | 112/13383 |
| GSE11352 [5] | Estradiol | (*H. sapiens* MCF7) | 0.05 | 114/20555 |

1^st^ column denotes GEO accession, 2^nd^ column is the chemical exposed to the samples. 4^th^ column is the median FDR for SAM. * denotes “high” dosage of Bisphenol A used for the exposed sample group.

**Additional File 1: Supplementary Table S2. Gene expression dataset summary for query stage**.

| **Dataset** | **Disease State** | **Number of Samples/Controls** | **SAM: median FDR** | **Number of Differentially Expressed Genes / Total** |
| --- | --- | --- | --- | --- |
| Chandran et al, GSE6919 [6] | Primary Prostate Cancer | 65/17 | 0.05 | 2989/16264 |
| Landi et al, GSE10072 [7] | Lung cancer (non-smokers) | 16/15 | 0.01 | 4494/13306 |
|  | Lung cancer (smokers) | 24/15 | 0.05 | 6067/13306 |
| Liu et al, GSE6883 [8] | Breast cancer (non-tumorigenic) | 3/3 | 0.05 | 48/13306 |
|  | Breast cancer (tumorigenic) | 6/3 | 0.05 | 259/13306 |

1^st^ column denotes GEO accession, 2^nd^ column is the disease state for affected samples. 4^th^ column is the median FDR for SAM.

**Additional File 1: Supplementary Table S3.** **Prediction of environmental factors** **associated with lung cancer smoker samples (GSE10072).**

| **Chemical Predicted** | **Hypergeo- metric P-value** | **Rank (percentile)** | **q-value** | **Relevant genes in set (number of references)** | **Citations** |
| --- | --- | --- | --- | --- | --- |
| Sodium arsenite | 1x10^-7^ | 19 (99) | 0 | JUN(13), HSPA1A(9), MAPK1(9) | [9-11] |
| Indomethacin | 5x10^-7^ | 20 (99) | 0 | PTGS(6), CCND1(4), BIRC5(3) | [12-14] |
| Dimethylnitrosamine | 1x10^-4^ | 32 (98) | 0.001 | ACTA2(19), TIMP1(15), PCNA(6) | [15] |
| Vanadium pentoxide | 1x10^-4^ | 33 (97) | 0.001 | HBEGF(3), CXCL10(2), MAPK1(2) | [16] |
| Bexarotene | 3x10^-4^ | 44 (97) | 0.004 | DUSP1(2), CCND(2) | [17] |

Shown in the table are a subset of the highly ranked factors (p < 0.01) that were predicted to have association with lung cancer gene expression (smokers) and had evidence of association with the MeSH term “Lung Neoplasms”. The 1^st^ column represents the factor predicted and the 2^nd^ and 3^rd^ columns show the hypergeometric p-value and ranking. The 4^th^ column shows q-value derived from random samples of genes. The 5^th^ column shows the notable genes in the chemical-gene set that were differentially expressed. The 6^th^ column contains references (see below) for the prostate cancer and chemical association found from the CTD.

**Additional File 1: Supplementary Table S4.** **Prediction of environmental factors** **associated with breast cancer samples (GSE6883).**

| **Chemical Predicted** | **Hypergeo- metric P-value** | **Rank (percentile)** | **q-value** | **Relevant genes in set (number of references)** | **Citations** |
| --- | --- | --- | --- | --- | --- |
| Benzene | 4x10^-6^ | 2 (100) | 0 | JUN(4), LPL(2), RGS2(2) | [18, 19] |
| Estradiol | 2x10^-4^ | 14 (99) | 0.008 | JUN(8), LPL(4), BCL2(3) | [20-25] |
| Progesterone | 1x10^-3^ | 19 (99) | 0.02 | LDLR(4), CLDN4(3), RGS2(3) | [26-28] |
| Tamoxifen | 2x10^-3^ | 23 (98) | 0.03 | F8(2), JUN(2), LPL(2) | [22, 29-31] |
| Resveratrol | 3x10^-3^ | 27 (98) | 0.05 | BCL2(9), JUN(4), JUND(2) | [32] |
| Fenretinide | 4x10^-3^ | 34 (97) | 0.07 | BCL2(3), CXCL2(2), ATF4(2) | [33] |

Shown in the table are a subset of the highly ranked factors (p < 0.01) that were predicted to have association with breast cancer gene expression (tumorigenic) and had evidence of association with the MeSH term “Breast Neoplasms”. Columns have similar definitions as Supplementary Table 3.


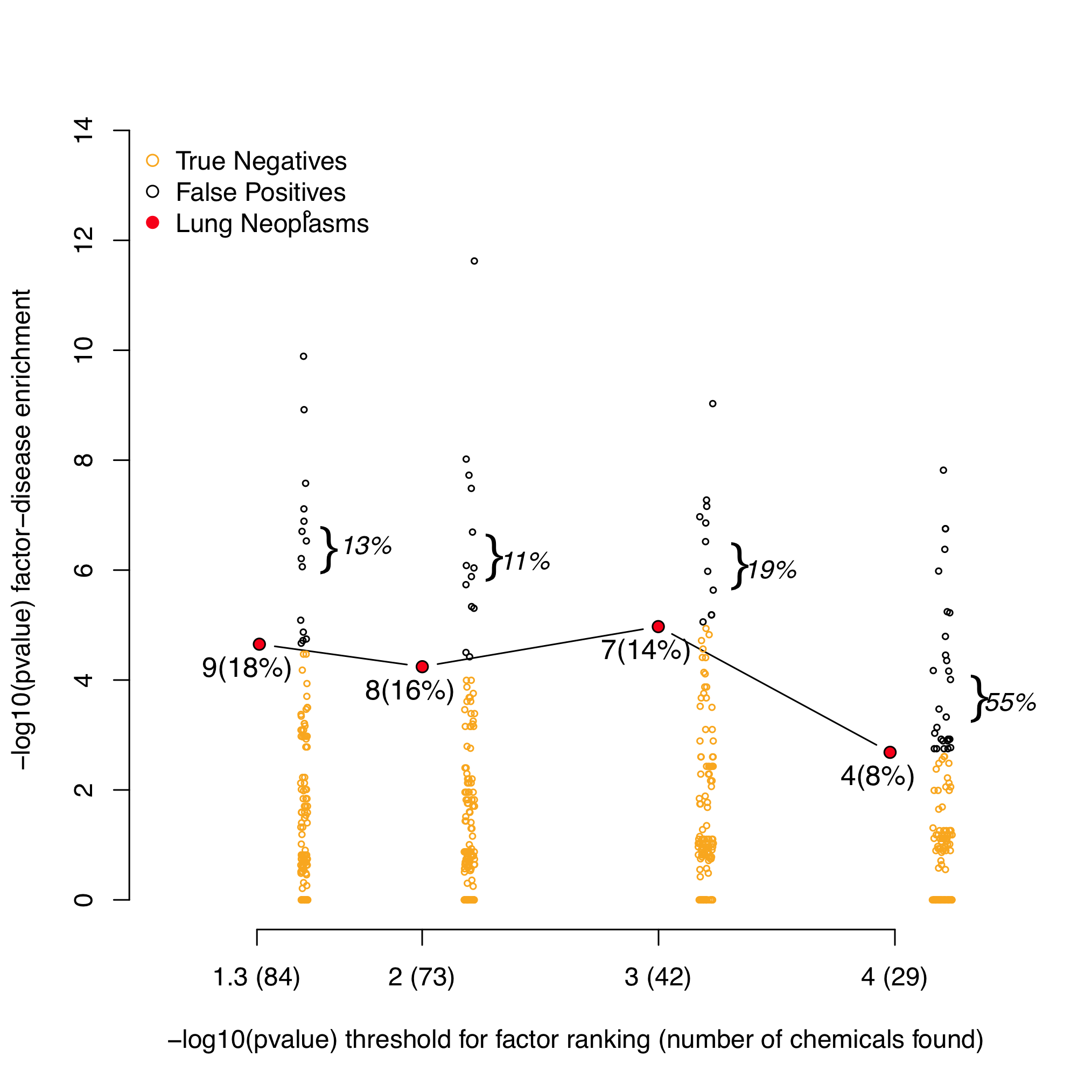


**Additional File 1: Supplementary** **Figure S1. Predicting environmental factor association to smoker, lung cancer datasets.** For a prediction list, we selected factors that ranked within α=10^-4^, 10^-3^, 10^-2^, and 0.05. This –log10(threshold) along with number of chemicals found (in parentheses) under each threshold is seen on the x-axis of each figure. We tested if these highly ranked factors found under each threshold were enriched for chemicals that had known curated association with the cancer in question. The –log10(p-value) for this enrichment is seen on the y-axis. The solid round red marker represents the enrichment test for the actual disease the predictions were based; the number underneath represents the number of chemicals found that had a curated association with the disease and the percent among all curated relations found. We estimated accuracy and precision by computing factor-disease enrichment for all other diseases; false positives are offset in black and true negatives are in yellow. The percentage of false positives are bracketed and in italics.

**
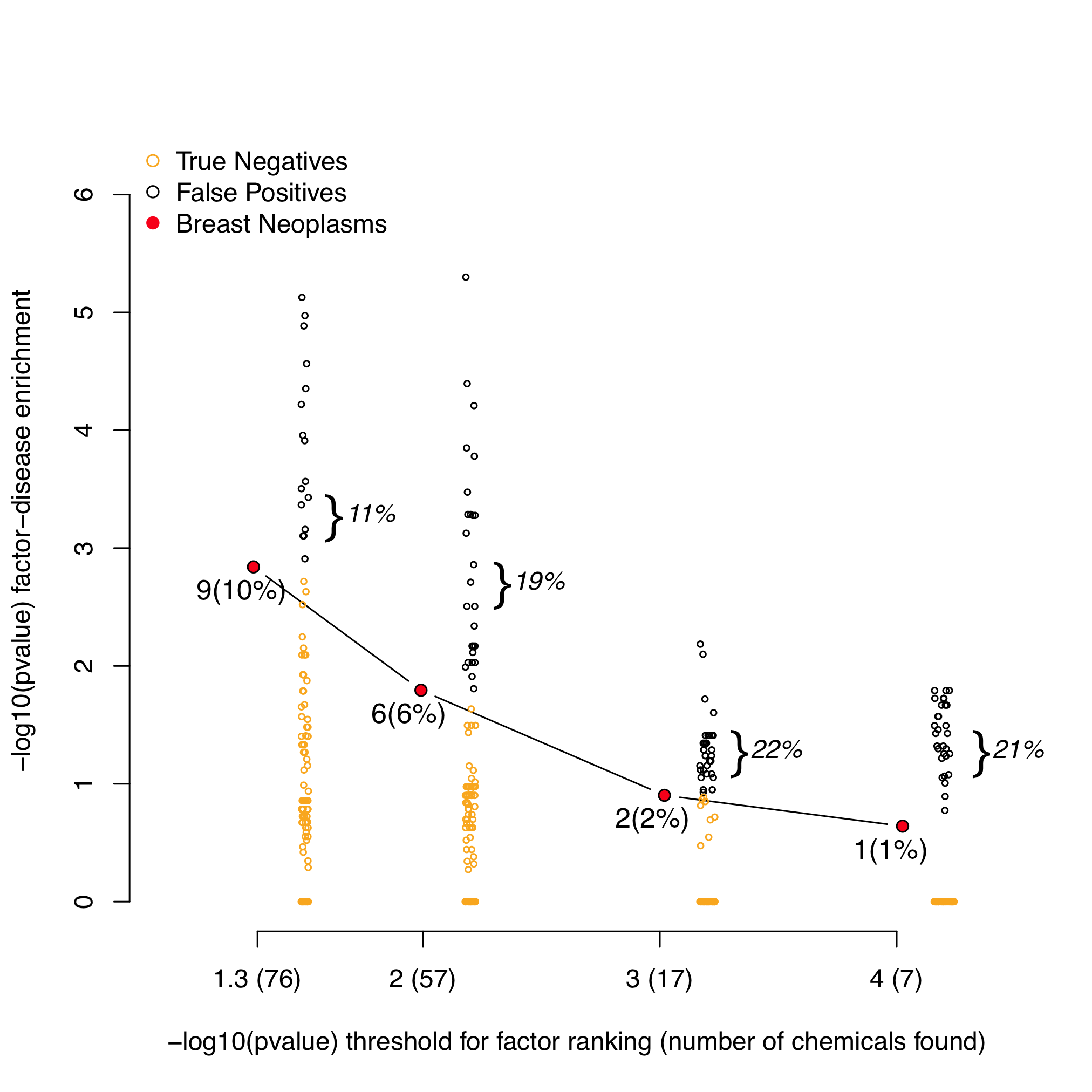
**

**Additional File 1: Supplementary** **Figure S2. Predicting environmental factor association to tumorigenic, breast cancer datasets.**

References:

1. Bossé Y, Maghni K, Hudson TJ: **1alpha,25-dihydroxy-vitamin D3 stimulation of bronchial smooth muscle cells induces autocrine, contractility, and remodeling processes**. *Physiol Genomics* 2007, **29**(2):161-168.

2. Tijet N, Boutros PC, Moffat ID, Okey AB, Tuomisto J, Pohjanvirta R: **Aryl hydrocarbon receptor regulates distinct dioxin-dependent and dioxin-independent gene batteries**. *Mol Pharmacol* 2006, **69**(1):140-153.

3. Li Z, Stonehuerner J, Devlin RB, Huang YC: **Discrimination of vanadium from zinc using gene profiling in human bronchial epithelial cells**. In: *Environ Health Perspect.* vol. 113; 2005: 1747-1754.

4. Selvaraj V, Bunick D, Finnigan-Bunick C, Johnson RW, Wang H, Liu L, Cooke PS: **Gene expression profiling of 17beta-estradiol and genistein effects on mouse thymus**. *Toxicol Sci* 2005, **87**(1):97-112.

5. Lin CY, Vega VB, Thomsen JS, Zhang T, Kong SL, Xie M, Chiu KP, Lipovich L, Barnett DH, Stossi F *et al*: **Whole-genome cartography of estrogen receptor alpha binding sites**. *PLoS Genet* 2007, **3**(6):e87.

6. Chandran UR, Ma C, Dhir R, Bisceglia M, Lyons-Weiler M, Liang W, Michalopoulos G, Becich M, Monzon FA: **Gene expression profiles of prostate cancer reveal involvement of multiple molecular pathways in the metastatic process**. *BMC Cancer* 2007, **7**:64.

7. Landi MT, Dracheva T, Rotunno M, Figueroa JD, Liu H, Dasgupta A, Mann FE, Fukuoka J, Hames M, Bergen AW *et al*: **Gene expression signature of cigarette smoking and its role in lung adenocarcinoma development and survival**. *PLoS ONE* 2008, **3**(2):e1651.

8. Liu R, Wang X, Chen GY, Dalerba P, Gurney A, Hoey T, Sherlock G, Lewicki J, Shedden K, Clarke MF: **The prognostic role of a gene signature from tumorigenic breast-cancer cells**. *N Engl J Med* 2007, **356**(3):217-226.

9. Shen J, Liu J, Xie Y, Diwan BA, Waalkes MP: **Fetal onset of aberrant gene expression relevant to pulmonary carcinogenesis in lung adenocarcinoma development induced by in utero arsenic exposure**. *Toxicol Sci* 2007, **95**(2):313-320.

10. Waalkes MP, Liu J, Ward JM, Diwan BA: **Enhanced urinary bladder and liver carcinogenesis in male CD1 mice exposed to transplacental inorganic arsenic and postnatal diethylstilbestrol or tamoxifen**. *Toxicol Appl Pharmacol* 2006, **215**(3):295-305.

11. Waalkes MP, Liu J, Ward JM, Diwan BA: **Animal models for arsenic carcinogenesis: inorganic arsenic is a transplacental carcinogen in mice**. *Toxicol Appl Pharmacol* 2004, **198**(3):377-384.

12. Diament MJ, Peluffo GD, Stillitani I, Cerchietti LC, Navigante A, Ranuncolo SM, Klein SM: **Inhibition of tumor progression and paraneoplastic syndrome development in a murine lung adenocarcinoma by medroxyprogesterone acetate and indomethacin**. *Cancer Invest* 2006, **24**(2):126-131.

13. Moody TW, Leyton J, Zakowicz H, Hida T, Kang Y, Jakowlew S, You L, Ozbun L, Zia H, Youngberg J *et al*: **Indomethacin reduces lung adenoma number in A/J mice**. *Anticancer Res* 2001, **21**(3B):1749-1755.

14. Levin G, Kariv N, Khomiak E, Raz A: **Indomethacin inhibits the accumulation of tumor cells in mouse lungs and subsequent growth of lung metastases**. *Chemotherapy* 2000, **46**(6):429-437.

15. Zanesi N, Mancini R, Sevignani C, Vecchione A, Kaou M, Valtieri M, Calin GA, Pekarsky Y, Gnarra JR, Croce CM *et al*: **Lung cancer susceptibility in Fhit-deficient mice is increased by Vhl haploinsufficiency**. *Cancer Res* 2005, **65**(15):6576-6582.

16. Devereux TR, Holliday W, Anna C, Ress N, Roycroft J, Sills RC: **Map kinase activation correlates with K-ras mutation and loss of heterozygosity on chromosome 6 in alveolar bronchiolar carcinomas from B6C3F1 mice exposed to vanadium pentoxide for 2 years**. *Carcinogenesis* 2002, **23**(10):1737-1743.

17. Alyaqoub FS, Liu Y, Tao L, Steele VE, Lubet RA, Pereira MA: **Modulation by bexarotene of mRNA expression of genes in mouse lung tumors**. *Mol Carcinog* 2008, **47**(3):165-171.

18. Houle CD, Ton TV, Clayton N, Huff J, Hong HH, Sills RC: **Frequent p53 and H-ras mutations in benzene- and ethylene oxide-induced mammary gland carcinomas from B6C3F1 mice**. *Toxicol Pathol* 2006, **34**(6):752-762.

19. Bennett LM, Davis BJ: **Identification of mammary carcinogens in rodent bioassays**. *Environ Mol Mutagen* 2002, **39**(2-3):150-157.

20. Lakshmanaswamy R, Guzman RC, Nandi S: **Hormonal prevention of breast cancer: significance of promotional environment**. *Adv Exp Med Biol* 2008, **617**:469-475.

21. Bergman Jungestrom M, Thompson LU, Dabrosin C: **Flaxseed and its lignans inhibit estradiol-induced growth, angiogenesis, and secretion of vascular endothelial growth factor in human breast cancer xenografts in vivo**. *Clin Cancer Res* 2007, **13**(3):1061-1067.

22. Vogel VG: **Recent results from clinical trials using SERMs to reduce the risk of breast cancer**. *Ann N Y Acad Sci* 2006, **1089**:127-142.

23. Eliassen AH, Missmer SA, Tworoger SS, Spiegelman D, Barbieri RL, Dowsett M, Hankinson SE: **Endogenous steroid hormone concentrations and risk of breast cancer among premenopausal women**. *J Natl Cancer Inst* 2006, **98**(19):1406-1415.

24. Russo J, Hasan Lareef M, Balogh G, Guo S, Russo IH: **Estrogen and its metabolites are carcinogenic agents in human breast epithelial cells**. *J Steroid Biochem Mol Biol* 2003, **87**(1):1-25.

25. Zou E, Matsumura F: **Long-term exposure to beta-hexachlorocyclohexane (beta-HCH) promotes transformation and invasiveness of MCF-7 human breast cancer cells**. *Biochem Pharmacol* 2003, **66**(5):831-840.

26. Rocha A, Azevedo I, Soares R: **Progesterone sensitizes breast cancer MCF7 cells to imatinib inhibitory effects**. *J Cell Biochem* 2008, **103**(2):607-614.

27. Carvajal A, Espinoza N, Kato S, Pinto M, Sadarangani A, Monso C, Aranda E, Villalon M, Richer JK, Horwitz KB *et al*: **Progesterone pre-treatment potentiates EGF pathway signaling in the breast cancer cell line ZR-75**. *Breast Cancer Res Treat* 2005, **94**(2):171-183.

28. Kato S, Pinto M, Carvajal A, Espinoza N, Monso C, Sadarangani A, Villalon M, Brosens JJ, White JO, Richer JK *et al*: **Progesterone increases tissue factor gene expression, procoagulant activity, and invasion in the breast cancer cell line ZR-75-1**. *J Clin Endocrinol Metab* 2005, **90**(2):1181-1188.

29. Swaby RF, Sharma CG, Jordan VC: **SERMs for the treatment and prevention of breast cancer**. *Rev Endocr Metab Disord* 2007, **8**(3):229-239.

30. Saeki T, Tsuruo T, Sato W, Nishikawsa K: **Drug resistance in chemotherapy for breast cancer**. *Cancer Chemother Pharmacol* 2005, **56 Suppl 1**:84-89.

31. Gielen SC, Burger CW, Kuhne LC, Hanifi-Moghaddam P, Blok LJ: **Analysis of estrogen agonism and antagonism of tamoxifen, raloxifene, and ICI182780 in endometrial cancer cells: a putative role for the epidermal growth factor receptor ligand amphiregulin**. *J Soc Gynecol Investig* 2005, **12**(7):e55-67.

32. Vyas S, Asmerom Y, De Leon DD: **Insulin-like growth factor II mediates resveratrol stimulatory effect on cathepsin D in breast cancer cells**. *Growth Factors* 2006, **24**(1):79-87.

33. Zanardi S, Serrano D, Argusti A, Barile M, Puntoni M, Decensi A: **Clinical trials with retinoids for breast cancer chemoprevention**. *Endocr Relat Cancer* 2006, **13**(1):51-68.
